# Summary data of serum concentrations of 32 persistent organic pollutants in young adults in relation to summary scores of persistent organic pollutants

**DOI:** 10.1016/j.dib.2019.103720

**Published:** 2019-03-07

**Authors:** Jose R. Suarez-Lopez, Myron D. Gross, Duk-Hee Lee

**Affiliations:** a Department of Family Medicine and Public Health, University of California, San Diego. 9500 Gilman Drive #0725, La Jolla, CA 92093 0725, USA; b Department of Laboratory Medicine and Pathology, University of Minnesota, MMC 609 Mayo 8609, 420 Delaware, Minneapolis, MN 55455, USA; c Department of Preventive Medicine, School of Medicine, Kyungpook National University, 101 Dongin-dong, Jung-gu, Daegu, 700 422, South Korea

## Abstract

This data article presents mean serum concentrations (wet weight and lipid standardized) of 32 persistent organic pollutants (POPs) detected in >75% of participants of the Coronary Artery Risk Development in Young Adults (CARDIA) study across levels of POPs scores, and their corresponding coefficients of determination. POPs scores were calculated as: A) the sum of each participant's log-transformed POPs concentrations (∑ of log Pops], or B) as the sum of the participants' log-transformed concentrations of each POP divided by the groups' standard deviation of the corresponding log-transformed POP (POPs summary score. Scores were calculated for both wet weight and lipid standardized concentrations and for all 32 POPs and for PCBs and organochlorine pesticides separately. POPs summary scores analyses were used in the article “Organochlorine pesticides and polychlorinated biphenyls (PCBs) in early adulthood and blood lipids over a 23-year follow-up” [Suarez-Lopez et al., 2018].

**Table:** Specifications table

Subject area	*Public Health*
More specific subject area	*Environmental Health*
Type of data	*Tables*
How data was acquired	*Gas chromatography isotope dilution high-resolution mass spectrometry*
Data format	*Analyzed data*
Experimental factors	*Cross-sectional analyses*
Experimental features	*Median concentrations (wet-weights and lipid standardized) of 32 persistent organic pollutants across categories of persistent organic pollutant scores and their corresponding coefficients of determination.*
Data source location	*United States of America: Birmingham, AL; Chicago, IL; Minneapolis, MN; and Oakland, CA*
Data accessibility	*Data is in the article*
Related research article	*J.R. Suarez-Lopez, C.G. Clemesha, M. Porta, M.D. Gross, D.*—*H. Lee, Organochlorine pesticides and polychlorinated biphenyls (PCBs) in early adulthood and blood lipids over a 23-year follow-up, Environ. Toxicol. Pharmacol. (2018)*[1]*.*

**Table:** 

**Value of the data** • POPs composite scores are used in epidemiologic studies to reduce the number of associations tested, thus reducing the potential for spurious findings, and because the general population is exposed to a mixture of POPs. • This article presents coefficients of determination (R^2^) between specific POPs and various POPs scores, thus providing further exposure information of our published work using POPs summary scores. • This article provides deeper insight on POPs summary scores and their relationships with specific POPs exposure levels. These data can be used by investigators who are conducting analyses using POPs score analyses and provides information to compare exposure levels between the CARDIA study and other studies.

## 1 Data

Data is presented in 6 tables providing information on wet weight concentrations and lipid-standardized concentrations (median, 25th – 75th percentile, p for trend and R^2^) of persistent organic pollutants (POPs) in serum across quartiles of various POPs summary variables including 32 POP summary score, 8 organochlorine pesticide (OCP) summary score, 23 polychlorinated biphenyl (PCB) summary score, and sum of 32 log-transformed POPs based on both wet-weight and lipid standardized values.

## 2 Experimental design, materials and methods

### 2.1 Participants

POPs in serum were measured in samples collected in 1985–1986 (year-2 of follow-up) of 180 men and women from a nested case-control study within the Coronary Artery Risk Development in Young Adults (CARDIA) study [1], [2], [3]. Participants with diabetes (cases) consisted of 50% of participants (n = 90). However, cases must not have been diagnosed with diabetes during at year 0 or 2 of follow-up. The remaining 90 participants were frequency matched to cases on body mass index (BMI) and were randomly selected from those who had fasting glucose levels below 100 mg/dL at follow-up years 0, 7, 10, 15, and 20, had not been diagnosed with diabetes before year 20 of follow-up, and were not receiving glucose-lowering medications. Additional details of recruitment and participant selection been described in detail [1], [3].

### 2.2 Measurements

POPs concentrations of serum samples collected in year 2 of follow-up were measured using solid-phase extraction and final determination using gas chromatography isotope dilution high-resolution mass spectrometry [4], [5]. Nine OCPs, 35 PCB congeners, 10 polybrominated diphenyl ether (PBDE) congeners, and 1 polybrominated biphenyl (PBB) congener were measured. Of these, 32 POPs that were detected in at least 75% of participants (8 OCPs, 23 PCBs, and PBB-153, Table. 1) were included in the present article. Non-detectable concentrations were replaced with 50% of the detection limit.

**Table 1:** Median (25th, 75th percentile) wet weight concentrations (pg/g) in serum across quartiles of 32 POP summary score. This list includes 32 POPs that were detectable in ≥75% of participants at year 2 of follow-up. Non-detectable values were replaced with 50% of the limit of detectability.

	All	Quartiles of 32 POP summary score				P-trend	R^2^
		Q1	Q2	Q3	Q4		
**Organochlorine pesticides**							
Hexachlorobenzene	154.1 (116.6–198.9)	112.3 (95, 151.6)	151.2 (115.9, 202.9)	161.1 (136.8, 203.7)	185.1 (149.4, 224.6)	<0.001	0.13
Mirex	24.0 (13.1–44.2)	13.2 (6.6, 21.9)	20.4 (7.2, 31)	29.8 (15.8, 49.7)	35.5 (22.8, 99.6)	<0.001	0.09
Oxychlordane	158.1 (116.4–209.7)	103.7 (82, 131.2)	135.8 (116.3, 164.6)	181.9 (152.5, 208.2)	237.6 (192.5, 337.1)	<0.001	0.56
p,p’-DDE	3478 (2357–5586)	1682 (1134, 2636)	3834 (2832, 5134)	3520 (2802, 5260)	6249 (3826, 9799)	<0.001	0.24
p,p’-DDT	132.7 (89.8–203.7)	90.9 (76.4, 130.2)	135.9 (96.3, 208.9)	136.3 (88.4, 185.6)	176.9 (124.5, 279.5)	<0.001	0.16
Trans-nonachlor	183.5 (127.5–263.8)	101.7 (82.1, 137.8)	156.8 (128.4, 197.9)	222.7 (167, 264.3)	316.9 (233.7, 481.8)	<0.001	0.47
β-hexachlorocyclohexane	69.5 (49.4–96.2)	45.8 (30.9, 58.2)	65.3 (50.2, 80.1)	77.1 (57.4, 98.9)	97.0 (75.7, 137.5)	<0.001	0.33
Λ-hexachlorocyclohexane	44.5 (18.1–121)	20.6 (16.4, 67.1)	57.1 (17.2, 160.4)	56.3 (23.7, 88.8)	58.8 (33.4, 208.4)	0.118	0.01
**PCBs**							
PCB74^a^	92.4 (62–145.3)	60.2 (44.3, 77.2)	91.6 (69.5, 119.2)	105.2 (68.2, 154.0)	144.1 (114.7, 183.5)	<0.001	0.39
PCB87	6.8 (3.5–10.2)	4.2 (2.2, 6.1)	6.7 (3.0, 8.5)	7.0 (5.0, 8.7)	11.5 (7.1, 15.1)	<0.001	0.22
PCB99	90.9 (60.5–136.7)	49.0 (37.8, 65.6)	85.2 (66.4, 115.3)	94.1 (80, 115.2)	160.0 (134.6, 218.6)	<0.001	0.56
PCB105^a^	29.8 (20.4–45.8)	17.6 (11.7, 27.1)	30.6 (21.9, 40.1)	28.6 (20.5, 42.3)	48.5 (36.5, 60.2)	<0.001	0.32
PCB118^a^	138 (92.4–212.9)	87.8 (58.1, 113.7)	146 (108.6, 185.6)	138.1 (108.7, 211.3)	231.5 (188.2, 282.0)	<0.001	0.39
PCB146	37.7 (25.2–61.1)	19.1 (13.2, 21.3)	33.4 (27.5, 38.2)	48.3 (37, 56.3)	83.5 (63.5, 109.6)	<0.001	0.61
PCB153	330.9 (220.8–496.8)	176.1 (146.3, 199.1)	290.9 (265.8, 328.9)	388.9 (351, 448.5)	628.0 (539.2, 783.4)	<0.001	0.72
PCB156^a^	42.5 (27.7–60.4)	23.1 (18.3, 27.7)	35.6 (27.6, 43.8)	48.6 (41.8, 59)	74.5 (60.9, 109.9)	<0.001	0.62
PCB157^a^	10.7 (7.1–15.4)	6.1 (4.8, 6.9)	8.5 (7.1, 11.7)	13.2 (10.1, 15.5)	18.8 (14.9, 25.8)	<0.001	0.62
PCB138-158	253.8 (187.6–362.7)	135.4 (106.8, 162.5)	224.3 (202.9, 266)	273.3 (253.4, 334.4)	468.9 (387.1, 592.7)	<0.001	0.67
PCB167^a^	13.5 (8.9–19.5)	7.3 (5.9, 8.6)	12.4 (9.4, 14)	15.3 (13.5, 18.9)	25.4 (19.1, 33)	<0.001	0.54
PCB170	77.8 (57.2–123.8)	41.5 (33.6, 49.4)	66.8 (59.1, 76.4)	96.8 (85.3, 110.5)	153.4 (130.3, 195.7)	<0.001	0.75
PCB177	19.6 (13.2–29.7)	9.1 (7.8, 11.5)	16.9 (15.3, 19.8)	22.2 (19.7, 25.1)	42.2 (32.9, 53.5)	<0.001	0.68
PCB178	13.8 (9.7–22.3)	6.7 (5.1, 8.9)	11.6 (9.8, 12.9)	16.3 (14.8, 21.7)	32.7 (23.1, 42.6)	<0.001	0.67
PCB180	205.1 (141.3–323.6)	105.8 (83.4, 128.2)	169.4 (144.5, 191.9)	244.1 (225.4, 299.7)	379.4 (334.1, 481)	<0.001	0.75
PCB183	30.9 (21.1–44.6)	16.5 (12.4, 18.7)	29.1 (23.9, 31.2)	36.3 (30.9, 40)	62.5 (48.3, 76.2)	<0.001	0.72
PCB187	74.4 (50.0–113.1)	34.8 (29.1, 45.2)	61.9 (52.9, 70.6)	86.4 (76.5, 109.2)	161.5 (115.2, 216.3)	<0.001	0.67
PCB194	41.3 (27.3–65.9)	20.4 (15.2, 25.9)	32.8 (27.9, 45.4)	50.6 (40.4, 64.7)	83.4 (66.4, 104.8)	<0.001	0.64
PCB195	11.5 (7.6–17.0)	5.8 (4.1, 6.7)	9.3 (7.9, 11.3)	13.1 (11.7, 15.5)	23.0 (18.0, 28.3)	<0.001	0.73
PCB199	44.4 (28.6–68.2)	23.3 (15.4, 28.6)	32.6 (28.6, 43.1)	50.6 (45.0, 66.7)	82.1 (68.3, 111.2)	<0.001	0.60
PCB196-203	47.5 (32.2–73.7)	24.6 (19, 29.9)	37.1 (33.2, 44.4)	54.3 (49.4, 67.0)	92.2 (75.8, 106.3)	<0.001	0.69
PCB206	23.0 (16.0–36.0)	13.0 (9.7, 17.0)	19.0 (15.5, 23.5)	28.0 (22.0, 36.0)	40.5 (34.5, 56.0)	<0.001	0.47
PCB209^a^	9.1 (6.0–14.7)	5.0 (3.8, 6.7)	6.9 (5.8, 9.4)	11.1 (8.4, 14.1)	17.7 (13.6, 23.2)	<0.001	0.47
**PBBs**							
PBB153	16.6 (10.9–24.2)	10.4 (6.6, 13.3)	12.5 (10.2, 22.4)	20.7 (15.8, 27.3)	21.4 (17, 28.8)	0.028	0.03

a Dioxin-like PCBs.

Total cholesterol, and triglyceride concentrations were measured using an enzymatic assay. HDL concentrations were measured via precipitation using dextran sulfate-magnesium chloride on the ABA 200 Biochromatic device. LDL concentrations were calculated using the Friedewald equation [6]. Oxidized LDL concentrations were measured using a monoclonal antibody mAb-4E6–based competition ELISA (Mercodia, Uppsala, Sweden).

### 2.3 POPs scores and analysis

This article presents median POPs concentrations in serum across categories of POPs composite variables. Composite variables are used because the general population is exposed to a mixture of many POPs [7], and such variables substantially reduce the number of associations tested, thus reducing the potential for spurious findings. First, we created a summary score which consisted of the sum of log-transformed POPs concentrations for each individual (∑ of 32 log POPs). Then we created composite POPs variables that would not be heavily influenced by the most prevalent POPs, considering that the 8 most prevalent POPs had 25.6 times the concentration than the 8 least prevalent in our study. This “32 POP summary score” was defined as the sum of the participants' log-transformed concentrations of each of the 32 POPs divided by the groups’ standard deviation of the corresponding log-transformed POP (Σ[logPOP_individual_/logPOP standard deviation_group_]). We created similar summary variables for 8 organochlorine pesticides (8 OCP summary score), and 23 PCBs (23 PCB summary score); summary variables were created for both wet weight and lipid standardized (POP concentration/total blood lipids) concentrations.

Table 1, Table 2, Table 3, Table 4, Table 5, Table 6 present the median and interquartile range of concentrations of each POP across quartiles of each of the POPs composite scores. Using linear regression, we calculated the p-trend for the associations between each POP concentration and POP composite score using continuous variables. We also calculated the coefficient of determination (R^2^, Pearson correlation) for each association.

**Table 2:** POPs median (25th, 75th percentile) lipid-standardized concentrations (pg/g) in serum across quartiles of 32 POP summary score (lipid standardized). This list includes 32 POPs that were detectable in ≥75% of participants at year 2 of follow-up. Non-detectable values were replaced with 50% of the limit of detectability.

			All	Quartiles of 32 POP summary score				P-trend	R^2^
				Q1	Q2	Q3	Q4		
**Organochlorine pesticides**									
Hexachlorobenzene			30.0 (23.8, 38.7)	26.3 (20.8, 30.4)	30.7 (22.4, 41.4)	31.1 (26.4, 38.1)	33.5 (25.7, 42.3)	0.001	0.06
Mirex			4.6 (2.3, 9.2)	2.3 (1.4, 4.4)	4.7 (3.0, 8.1)	4.6 (2.5, 8.1)	8.1 (4.4, 12.1)	0.001	0.06
Oxychlordane			31.5 (24.5, 40.9)	22.2 (18.7, 28.4)	29.1 (23.7, 31.6)	37.0 (30.5, 42.2)	43.3 (37.5, 50.9)	<0.001	0.46
p,p’-DDE			723.3 (455.8, 1150.4)	434.5 (240.1, 580.9)	641.5 (462.9, 886.9)	923.6 (555.6, 1436.7)	955.7 (752.8, 1566.8)	<0.001	0.18
p,p’-DDT			26.1 (19.6, 38.7)	21.1 (14.8, 28.7)	25.9 (20.1, 38.5)	27.0 (20.1, 45.7)	28.1 (21.6, 50.1)	<0.001	0.10
Trans-nonachlor			36.7 (26.5, 50.6)	22.9 (19.3, 30)	32.2 (26.5, 39.9)	43.3 (33.5, 49.7)	58.6 (43.6, 72.1)	<0.001	0.37
β-hexachlorocyclohexane			13.7 (9.9, 18.4)	9.9 (7.0, 13.7)	11.9 (9.8, 14.9)	14.9 (12.0, 21.3)	18.1 (15.5, 25.9)	<0.001	0.26
Λ-hexachlorocyclohexane			8.9 (3.7, 24.4)	5.4 (3.4, 14.1)	9.5 (3.5, 25.7)	10.1 (4.1, 18.2)	10.6 (5.0, 40.6)	0.258	0.01
**PCBs**									
PCB74			3.1 (1.4, 4.6)	2.8 (1.4, 4)	2.7 (1.3, 4.9)	3.0 (1.4, 5.5)	3.8 (2.1, 5.3)	<0.001	0.11
PCB87			0.8 (0.5, 1.3)	0.6 (0.5, 1.1)	0.9 (0.5, 1.6)	0.9 (0.5, 1.3)	0.8 (0.5, 1.7)	0.003	0.05
PCB99			1.3 (0.8, 2.0)	0.9 (0.5, 1.4)	1.3 (0.8, 1.9)	1.6 (0.8, 2.0)	1.6 (1.1, 2.7)	<0.001	0.16
PCB105			0.6 (0.3, 1.1)	0.3 (0.3, 0.6)	0.6 (0.3, 0.9)	0.7 (0.3, 1.0)	1.2 (0.6, 1.9)	<0.001	0.23
PCB118			6.2 (3.8, 8.6)	4.6 (2.7, 6.3)	5.6 (3.4, 8.1)	6.8 (4.3, 9.0)	8.3 (6, 11.4)	<0.001	0.30
PCB146			67.7 (46.4, 92.2)	38.7 (32.0, 41.6)	57.3 (51.7, 66.5)	76.7 (68.2, 88.7)	118.5 (95.1, 143.1)	<0.001	0.79
PCB153			49.5 (37.2, 72.7)	27.9 (23.9, 37.2)	45.2 (37.5, 49.6)	55.9 (48.9, 63.5)	85.6 (73.0, 111.6)	<0.001	0.73
PCB156			2.0 (1.4, 3.1)	1.2 (1.0, 1.5)	1.7 (1.4, 2.2)	2.2 (2.0, 3.2)	3.8 (2.7, 4.3)	<0.001	0.59
PCB157			2.9 (1.9, 4.4)	1.5 (1.1, 1.8)	2.3 (2.0, 2.8)	3.2 (2.9, 4.3)	5.5 (4.4, 7.9)	<0.001	0.70
PCB138-158			0.6 (0.3, 1.5)	0.3 (0.3, 0.8)	0.9 (0.3, 1.6)	0.7 (0.3, 1.4)	1.5 (0.3, 2.2)	<0.001	0.11
PCB167			8.2 (5.8, 12.1)	4.8 (4.1, 6.4)	6.8 (5.7, 8.4)	9.2 (8.0, 12.1)	14.0 (11.4, 16.6)	<0.001	0.58
PCB170			0.4 (0.3, 0.8)	0.3 (0.2, 0.4)	0.4 (0.3, 0.6)	0.6 (0.3, 0.9)	1.0 (0.7, 1.3)	<0.001	0.41
PCB177			1.6 (0.3, 2.8)	0.4 (0.3, 1.2)	1.4 (0.2, 1.8)	2.2 (0.3, 2.6)	3.6 (3.0, 5.1)	<0.001	0.52
PCB178			14.7 (9.8, 21.4)	7.5 (6.2, 8.8)	12.6 (11.1, 15)	18.0 (14.8, 20.9)	29.0 (22.8, 36.4)	<0.001	0.72
PCB180			15.8 (11.5, 23.4)	8.7 (7.8, 10.6)	14.0 (12.2, 15.1)	19.8 (16.2, 23.6)	28.6 (23.4, 34.7)	<0.001	0.75
PCB183			3.8 (2.8, 5.7)	2.0 (1.6, 2.4)	3.4 (3.0, 3.8)	4.7 (3.8, 5.6)	7.5 (5.3, 8.7)	<0.001	0.71
PCB187			6.1 (4.5, 8.8)	3.5 (2.9, 4.1)	5.5 (4.8, 6.3)	7.0 (5.8, 8.5)	10.7 (9.4, 13.4)	<0.001	0.77
PCB194			4.6 (3.3, 6.9)	2.8 (2.2, 3.4)	4.1 (3.1, 5.4)	5.4 (4.2, 7.0)	7.8 (5.8, 9.9)	<0.001	0.4
PCB195			8.4 (5.7, 12.0)	4.4 (3.6, 5.6)	7.5 (5.7, 9.2)	9.3 (7.6, 13.2)	14.5 (11.4, 19.4)	<0.001	0.59
PCB199			9.6 (6.5, 13.4)	5.2 (4.7, 6.3)	8.2 (7.0, 9.6)	11.1 (9.6, 12.8)	14.9 (13.5, 21.0)	<0.001	0.67
PCB196-203			2.3 (1.5, 3.3)	1.2 (0.9, 1.5)	1.8 (1.6, 2.3)	2.6 (2.3, 3.3)	3.8 (3.4, 4.9)	<0.001	0.72
PCB206			1.8 (1.2, 2.7)	1.1 (0.8, 1.3)	1.5 (1.1, 2.0)	2.1 (1.7, 3.0)	3.0 (2.5, 4.0)	<0.001	0.38
PCB209			0.28 (0.23, 0.32)	0.28 (0.24, 0.33)	0.26 (0.23, 0.32)	0.29 (0.24, 0.32)	0.26 (0.22, 0.31)	0.751	0.00
**PBBs**									
PBB153			3.1 (2.2, 4.8)	2.2 (1.6, 3.2)	2.7 (2.0, 3.9)	4.1 (3, 5.8)	3.9 (2.8, 5.5)	0.04	0.02

**Table 3:** POPs median (25th, 75th percentile) wet-weight concentrations (pg/g) in serum across quartiles of ∑ of 32 log POPs (based on wet weight concentrations). This list includes 32 POPs that were detectable in ≥75% of participants at year 2 of follow-up. Non-detectable values were replaced with 50% of the limit of detectability.

	Quartiles of ∑ of 32 log POPs (wet weight concentrations)				P-trend	R^2^
	Q1	Q2	Q3	Q4		
**Organochlorine pesticides**						
Hexachlorobenzene	112.3 (95.0, 151.6)	152.6 (119.4, 206.4)	159.2 (134.8, 203.7)	171.0 (149.4, 222.4)	<0.001	0.13
Mirex	13.2 (6.5, 21.9)	19.4 (7.2, 31.0)	30.1 (15.8, 44.3)	42.5 (22.8, 100.0)	<0.001	0.10
Oxychlordane	103.7 (82, 131.2)	139.4 (116.3, 164.6)	181.9 (155.2, 208.2)	237.6 (192.5, 337.1)	<0.001	0.54
p,p’-DDE	1682 (1134, 2636)	3764 (2632, 5063)	3538 (2902, 5611)	6249 (4025, 9799)	<0.001	0.25
p,p’-DDT	90.9 (76.4, 130.2)	128.1 (93.9, 204.8)	142.3 (94.1, 185.6)	176.9 (124.5, 279.6)	<0.001	0.16
Trans-nonachlor	101.7 (82.1, 137.8)	156.8 (128.4, 197.9)	220.4 (167, 256.4)	316.9 (239.5, 481.8)	<0.001	0.47
β-hexachlorocyclohexane	45.79 (30.91, 58.22)	65.3 (50.17, 80.31)	76.89 (62.08, 98.85)	96.99 (75.67, 137.49)	<0.001	0.33
Λ-hexachlorocyclohexane	20.6 (16.4, 67.1)	40.2 (15.4, 160.3)	57.3 (29.2, 88.8)	65.5 (33.4, 211.6)	0.04	0.02
**PCBs**						
PCB74	60.2 (44.3, 77.2)	91.9 (73.2, 124.5)	105.2 (68.2, 154)	144.1 (114.7, 183.5)	<0.001	0.36
PCB87	4.2 (2.15, 6.1)	6.5 (1.4, 8.3)	7.3 (5.7, 9)	11.2 (6.3, 15.1)	<0.001	0.22
PCB99	49.0 (37.8, 65.6)	85.2 (66.4, 111.8)	95.8 (80.4, 122.9)	160.0 (134.6, 218.6)	<0.001	0.54
PCB105	17.6 (11.7, 27.1)	28.8 (21.9, 40.9)	31.5 (20.7, 42.3)	48.5 (35.1, 60.2)	<0.001	0.3
PCB118	87.8 (58.1, 113.7)	143.8 (108.6, 185.6)	144.4 (108.7, 211.3)	231.5 (188.2, 282)	<0.001	0.37
PCB146	19.1 (13.2, 21.3)	33.4 (27.5, 38.7)	48.3 (36.7, 55.8)	83.5 (63.8, 109.6)	<0.001	0.59
PCB153	176.1 (146.3, 199.1)	290.9 (265.8, 329.5)	388.9 (345.8, 461.7)	628.0 (539.2, 783.4)	<0.001	0.71
PCB156	23.1 (18.3, 27.7)	36.2 (28.3, 46)	48.6 (40.8, 59)	74.5 (58.6, 109.9)	<0.001	0.6
PCB157	6.1 (4.8, 6.9)	8.5 (7.2, 11.8)	12.0 (9.7, 15.5)	18.8 (14.6, 25.8)	<0.001	0.6
PCB138-158	135.4 (106.8, 162.5)	227.1 (202.9, 267.5)	273.3 (253.4, 335.8)	468.9 (387.1, 592.7)	<0.001	0.66
PCB167	7.3 (5.9, 8.6)	12.4 (9.4, 14.4)	15.4 (13, 18.9)	25.4 (19.1, 33)	<0.001	0.52
PCB170	41.5 (33.6, 49.4)	67.6 (59.9, 76.8)	96.8 (85.2, 110.5)	153.4 (130.6, 195.7)	<0.001	0.74
PCB177	9.1 (7.8, 11.5)	16.8 (15.2, 19.8)	22.2 (19.7, 24.7)	42.2 (32.9, 53.5)	<0.001	0.67
PCB178	6.7 (5.1, 8.9)	11.7 (9.8, 13.5)	16.0 (14.0, 21.5)	32.7 (23.6, 42.6)	<0.001	0.66
PCB180	105.8 (83.4, 128.2)	171.5 (148.0, 203.3)	244.0 (212.6, 299.7)	379.4 (339.8, 481)	<0.001	0.74
PCB183	16.5 (12.4, 18.7)	28.5 (23.9, 30.8)	36.3 (31.0, 41.5)	62.5 (48.3, 76.2)	<0.001	0.72
PCB187	34.8 (29.1, 45.2)	61.9 (52.9, 74.9)	85.4 (73.8, 108.6)	161.5 (116.3, 216.3)	<0.001	0.66
PCB194	20.4 (15.2, 25.9)	34.1 (27.9, 46.1)	47.9 (39.9, 61.5)	83.4 (66.4, 104.8)	<0.001	0.63
PCB195	5.8 (4.1, 6.7)	9.5 (8.3, 11.4)	12.9 (11.4, 15.2)	23.0 (18.1, 28.3)	<0.001	0.72
PCB199	23.3 (15.4, 28.6)	35.8 (28.6, 44.7)	48.7 (44.2, 65.4)	82.1 (69.7, 111.2)	<0.001	0.6
PCB196-203	24.6 (19, 29.9)	37.1 (33.2, 47.3)	53.5 (47.6, 62.4)	92.2 (76.9, 106.3)	<0.001	0.69
PCB206	13.0 (9.7, 17.0)	19.5 (16.0, 24.5)	27.0 (21.0, 34.0)	40.5 (35.0, 56.0)	<0.001	0.47
PCB209	5.0 (3.8, 6.7)	7.2 (5.8, 9.7)	11.0 (8.1, 13.8)	17.8 (13.7, 23.2)	<0.001	0.47
**PBBs**						
PBB153	10.4 (6.6, 13.3)	12.8 (10.2, 22.4)	20.7 (15.8, 27.3)	22.4 (17, 28.8)	0.03	0.03

**Table 4:** POPs median (25th, 75th percentile) lipid-standardized concentrations (pg/g) in serum across quartiles of ∑ of 32 log POPs (based on lipid-standardized concentrations). This list includes 32 POPs that were detectable in ≥75% of participants at year 2 of follow-up. Non-detectable values were replaced with 50% of the limit of detectability.

	Quartiles of ∑ of 32 log POPs (lipid standardized concentrations)				P-trend	R-square
	Q1	Q2	Q3	Q4		
**Organochlorine pesticides**						
Hexachlorobenzene	26.3 (20.8, 30.4)	30.4 (22.4, 37)	32.4 (26.9, 43.5)	33.2 (25.7, 42)	0.001	0.06
Mirex	2.3 (1.4, 4.2)	5.2 (3, 8.9)	4.5 (1.8, 8)	8.7 (4.7, 16.3)	<0.001	0.07
Oxychlordane	23.3 (18.7, 28.5)	28.2 (23.7, 32.5)	35.9 (29.3, 41.8)	42.8 (37.5, 50.9)	<0.001	0.42
P,p’-DDE	405.1 (240.1, 564.6)	662.4 (477.6, 886.9)	959.9 (555.6, 1458.0)	923.6 (752.8, 1566.8)	<0.001	0.20
P,p’-DDT	21.6 (14.8, 28.7)	24.5 (19.6, 37.5)	27 (20.1, 38.5)	31.4 (22.0, 51.2)	<0.001	0.11
Trans-nonachlor	23.6 (19.3, 30.0)	33.4 (25.6, 43)	40.6 (32.5, 49.7)	58.6 (43.6, 72.1)	<0.001	0.35
β-hexachlorocyclohexane	9.3 (6.6, 13.8)	12.3 (10.4, 14.8)	14.9 (11.2, 21.3)	17.8 (15.1, 25.9)	<0.001	0.27
Λ-hexachlorocyclohexane	4.5 (3.3, 11.2)	7.9 (3.4, 17.3)	11.9 (4.5, 34)	12.2 (5.9, 41.9)	0.061	0.02
**PCBs**						
PCB74	2.4 (1.4, 4)	2.9 (1.5, 4.9)	3.0 (1.4, 4.4)	3.8 (1.6, 5.3)	<0.001	0.11
PCB87	0.6 (0.4, 1.1)	0.9 (0.5, 1.2)	0.9 (0.5, 1.5)	0.8 (0.5, 1.7)	0.002	0.05
PCB99	0.9 (0.5, 1.4)	1.3 (0.8, 1.9)	1.6 (0.9, 2.1)	1.6 (1.1, 2.7)	<0.001	0.16
PCB105	0.3 (0.3, 0.6)	0.5 (0.3, 0.9)	0.6 (0.3, 1.0)	1.2 (0.7, 1.9)	<0.001	0.24
PCB118	4.1 (2.7, 6.3)	5.8 (3.4, 8.1)	7.3 (4.4, 9.0)	7.7 (5.8, 11.4)	<0.001	0.30
PCB146	38.7 (32.0, 41.6)	55.8 (51.7, 64.8)	76.5 (68.2, 88.7)	118.5 (92.8, 143.1)	<0.001	0.77
PCB153	27.9 (23.9, 36.8)	43.3 (38.5, 48.9)	59.3 (50.6, 71.3)	85.5 (72.4, 111.6)	<0.001	0.72
PCB156	1.2 (1.0, 1.5)	1.7 (1.4, 2.2)	2.2 (1.9, 3.2)	3.7 (2.6, 4.3)	<0.001	0.55
PCB157	1.5 (1.1, 1.8)	2.4 (2.1, 3.0)	3.2 (2.8, 4.3)	5.5 (4.4, 7.9)	<0.001	0.68
PCB138-158	0.3 (0.3, 0.8)	0.8 (0.3, 1.4)	0.7 (0.3, 1.4)	1.5 (0.3, 2.2)	<0.001	0.11
PCB167	4.8 (4.1, 6.4)	6.8 (5.7, 8.6)	9.5 (7.9, 12.2)	13.8 (11.3, 16.6)	<0.001	0.55
PCB170	0.3 (0.2, 0.4)	0.4 (0.3, 0.6)	0.6 (0.3, 0.9)	1.0 (0.7, 1.3)	<0.001	0.39
PCB177	0.7 (0.3, 1.2)	0.3 (0.2, 1.8)	2.1 (0.4, 2.6)	3.6 (2.8, 5.1)	<0.001	0.49
PCB178	7.5 (6.2, 8.8)	12.6 (11.0, 14.5)	18.0 (15.1, 20.8)	29.0 (23.1, 36.4)	<0.001	0.72
PCB180	8.7 (7.8, 10.6)	13.9 (11.9, 14.8)	18.5 (16.1, 22.3)	28.6 (23.4, 34.7)	<0.001	0.72
PCB183	2.1 (1.6, 2.5)	3.4 (3.0, 3.8)	4.8 (3.8, 5.6)	7.5 (5.7, 8.7)	<0.001	0.71
PCB187	3.5 (2.9, 4.2)	5.5 (4.8, 6.2)	7.1 (6, 8.6)	10.7 (9.4, 13.4)	<0.001	0.77
PCB194	2.8 (2.2, 3.5)	4.1 (3.1, 5.2)	5.4 (4.2, 6.9)	7.8 (6.0, 9.9)	<0.001	0.38
PCB195	4.4 (3.6, 5.7)	7.4 (5.7, 9.2)	9.3 (7.6, 12.4)	14.5 (11.5, 19.4)	<0.001	0.56
PCB199	5.2 (4.7, 6.3)	8.0 (6.6, 9.6)	11.1 (9.6, 12.8)	14.9 (13.5, 21)	<0.001	0.64
PCB196-203	1.2 (0.9, 1.5)	1.8 (1.6, 2.3)	2.7 (2.2, 3.1)	3.9 (3.4, 4.9)	<0.001	0.70
PCB206	1.1 (0.8, 1.3)	1.7 (1.1, 2.0)	2.0 (1.6, 2.8)	3.1 (2.5, 4.0)	<0.001	0.36
PCB209	0.3 (0.2, 0.3)	0.3 (0.2, 0.3)	0.3 (0.2, 0.3)	0.3 (0.2, 0.3)	0.827	0.00
**PBBs**						
PBB153	2.2 (1.6, 3.2)	2.9 (2.1, 5.4)	3.8 (2.7, 5.1)	3.9 (2.8, 5.5)	0.148	0.01

**Table 5:** OCP median (25th, 75th percentile) wet weight concentrations (pg/g) in serum across quartiles of 8 OCP summary score. This list includes 8 OCPs that were detectable in ≥75% of participants at year 2 of follow-up. Non-detectable values were replaced with 50% of the limit of detectability.

	Quartiles of 8 OCP summary score				P-trend	R-square
	Q1	Q2	Q3	Q4		
**Organochlorine pesticides**						
Hexachlorobenzene	112.9 (98.7, 151.6)	159 (125.1, 191.5)	148.8 (120.9, 203.6)	196.7 (154.9, 241.2)	<0.001	0.16
Mirex	6.8 (6.6, 20.2)	20.7 (13.2, 31)	30.2 (21.5, 54)	42.9 (22.9, 76.8)	<0.001	0.12
Oxychlordane	110.5 (87.3, 131.2)	152.4 (126.8, 181.9)	172.9 (135.8, 217.8)	234 (188.3, 338.6)	<0.001	0.46
P,p’-DDE	1831 (1134, 2513)	2954 (2346, 3928)	4025 (3021, 5561)	7072 (4800, 9799)	<0.001	0.31
P,p’-DDT	89.8 (72.1, 123)	123.3 (85.7, 155.7)	153.5 (104.0, 218.4)	228.0 (157.7, 319.4)	<0.001	0.25
Trans-nonachlor	101.7 (82.1, 151.3)	165 (126.3, 205.1)	205.5 (156.8, 263.3)	335.2 (250.3, 488.0)	<0.001	0.43
β-hexachlorocyclohexane	44.4 (29.6, 62.4)	58.2 (48.9, 72.1)	79.9 (61.4, 98.9)	107.4 (82.5, 140.4)	<0.001	0.42
Λ-hexachlorocyclohexane	17.9 (7.0, 40.3)	32.0 (18.1, 99.3)	55.7 (34.2, 102.1)	115.2 (43.9, 395.3)	<0.001	0.07

**Table 6:** PCB median (25th, 75th percentile) wet weight concentrations (pg/g) in serum across quartiles of 23 PCB summary score. This list includes 23 PCBs that were detectable in ≥75% of participants at year 2 of follow-up. Non-detectable values were replaced with 50% of the limit of detectability.

	Quartiles of 23 PCB summary score (wet weight concentrations)				P-trend	R-square
	Q1	Q2	Q3	Q4		
**Organochlorine pesticides**						
PCB74	60.2 (45.4, 73.3)	91.6 (73.2, 117.8)	113.6 (72.0, 161.1)	144.1 (114.7, 183.5)	<0.001	0.41
PCB87	4.2 (1.9, 6.3)	6.7 (3.9, 8.5)	7.1 (5.0, 10.6)	10.6 (6.3, 15.1)	<0.001	0.23
PCB99	49.0 (37.8, 65.5)	83.5 (63.8, 107.9)	96.0 (80.4, 129.2)	154.0 (134.3, 218.6)	<0.001	0.57
PCB105	17.1 (11.7, 27.1)	28.4 (20.3, 37.6)	34.6 (20.8, 49.3)	46.5 (32.8, 60.2)	<0.001	0.34
PCB118	86.1 (58.1, 125.8)	134.6 (93.5, 172.3)	166.5 (118.8, 225.0)	231.5 (182.9, 282.0)	<0.001	0.41
PCB146	19.1 (13.2, 21.3)	32.7 (27.5, 37.0)	48.3 (41.2, 55.8)	83.9 (64.0, 109.6)	<0.001	0.64
PCB153	176.1 (146.3, 195.2)	290.1 (261.1, 313.8)	388.9 (353.1, 445.9)	656.4 (552.1, 783.4)	<0.001	0.74
PCB156	21.5 (18.3, 27)	32.6 (28.0, 39.5)	50.0 (45.6, 59.8)	77.4 (65.5, 110.3)	<0.001	0.65
PCB157	5.6 (4.8, 6.9)	8.4 (7.3, 10.5)	13.4 (10.7, 15.6)	19.0 (15.1, 26.3)	<0.001	0.65
PCB138-158	135.4 (106.8, 161.9)	224.3 (203.9, 253.2)	296.5 (255.5, 349.3)	468.9 (388.1, 592.7)	<0.001	0.69
PCB167	6.9 (5.5, 8.9)	12.2 (9.4, 13.7)	17.1 (13.5, 19.6)	25.4 (18.5, 33.0)	<0.001	0.57
PCB170	41.5 (33.6, 50.3)	67.1 (57.7, 76.7)	96.8 (78.2, 110.0)	163.9 (133.5, 195.7)	<0.001	0.76
PCB177	9.1 (7.8, 11.4)	16.3 (15, 19.4)	22.2 (19.7, 24.5)	42.2 (32.9, 53.5)	<0.001	0.70
PCB178	6.5 (4.4, 8.8)	11.7 (10.0, 13.0)	16.4 (14.8, 21.3)	32.7 (25.0, 42.6)	<0.001	0.70
PCB180	105.8 (83.4, 123.6)	173.9 (143.8, 198.8)	244.1 (224.7, 275.7)	395.1 (347.3, 481.0)	<0.001	0.77
PCB183	16.5 (12.4, 19.4)	28.1 (23.2, 32.1)	35.2 (29.5, 40)	62.5 (47.8, 76.2)	<0.001	0.73
PCB187	36.0 (29.1, 45.6)	62.7 (53.9, 71.1)	86.4 (75.6, 103.1)	161.5 (124.3, 216.3)	<0.001	0.69
PCB194	20.0 (15.2, 25.9)	34.8 (28.2, 43.6)	50.3 (39.9, 61.5)	86.1 (66.7, 104.8)	<0.001	0.65
PCB195	5.7 (4.0, 6.8)	9.5 (8.4, 11.4)	13.3 (11.5, 15.2)	23.0 (18.1, 28.3)	<0.001	0.75
PCB199	22.8 (15.4, 28.0)	37.4 (29.8, 44.7)	50.6 (44.2, 65.8)	85.7 (69.8, 107.7)	<0.001	0.62
PCB196-203	24.6 (19.0, 29.9)	39.1 (34.6, 47.3)	55.0 (47.6, 67)	92.2 (77.2, 105.7)	<0.001	0.70
PCB206	13.0 (9.7, 16.0)	20.0 (16.0, 24.0)	28.0 (22.0, 34.0)	41.0 (35.0, 56.0)	<0.001	0.48
PCB209	5.0 (3.8, 6.3)	7.2 (5.9, 9.7)	10.9 (8.1, 14.1)	17.7 (13.6, 23.2)	<0.001	0.46

## References

[1] Suarez-Lopez J.R., Clemesha C.G., Porta M., Gross M.D., Lee D.-H. (2018). Organochlorine pesticides and polychlorinated biphenyls (PCBs) in early adulthood and blood lipids over a 23-year follow-up. Environ. Toxicol. Pharmacol..

[2] Lee D.-H., Steffes M.W., Sjödin A., Jones R.S., Needham L.L., Jacobs D.R. (2010). Low dose of some persistent organic pollutants predicts type 2 diabetes: a nested case-control study. Environ. Health Perspect..

[3] Suarez-Lopez J.R., Lee D.-H., Porta M., Steffes M.W., Jacobs D.R. (2015). Persistent organic pollutants in young adults and changes in glucose related metabolism over a 23-year follow-up. Environ. Res..

[4] Sjödin A., Jones R.S., Lapeza C.R., Focant J.-F., McGahee E.E., Patterson D.G. (2004). Semiautomated high-throughput extraction and cleanup method for the measurement of polybrominated diphenyl ethers, polybrominated biphenyls, and polychlorinated biphenyls in human serum. Anal. Chem..

[5] Barr J.R., Maggio V.L., Barr D.B., Turner W.E., Sjödin A., Sandau C.D., Pirkle J.L., Needham L.L., Patterson D.G. (2003). New high-resolution mass spectrometric approach for the measurement of polychlorinated biphenyls and organochlorine pesticides in human serum. J. Chromatogr. B. Analyt. Technol. Biomed. Life Sci..

[6] Friedewald W.T., Levy R.I., Fredrickson D.S. (1972). Estimation of the concentration of low-density lipoprotein cholesterol in plasma, without use of the preparative ultracentrifuge. Clin. Chem..

[7] Porta M., Puigdomènech E., Ballester F., Selva J., Ribas-Fitó N., Llop S., López T. (2008). Monitoring concentrations of persistent organic pollutants in the general population: the international experience. Environ. Int..

